# *Quality Improvement in Itself Changes Your Thinking*: Lessons From Disseminating Quality Improvement Methods Through a Multisite International Collaborative Palliative Care Project in India

**DOI:** 10.1200/GO.22.00147

**Published:** 2022-10-17

**Authors:** Aanchal Satija, Karl A. Lorenz, Odette Spruijt, Archana Ganesh, Nainwant Singh, Natalie B. Connell, Raziel C. Gamboa, Soraya Fereydooni, Shivani Chandrashekaran, Tayler Hennings, Karleen F. Giannitrapani, Sushma Bhatnagar

**Affiliations:** 1 Department of Onco-Anesthesia and Palliative Medicine, Dr B. R. Ambedkar, IRCH, AIIMS, New Delhi, India; 2 VA HSR&D Center for Innovation to Implementation (Ci2i), Menlo Park, CA; 3 Division of Primary Care and Population Health, Stanford University School of Medicine, Palo Alto, CA; 4 Peter MacCallum Cancer Center, University of Melbourne, Melbourne, VIC, Australia; 5 Yale School of Medicine, New Haven, CT; 6 Duke University School of Medicine, Durham, NC

## Abstract

Seven major palliative care (PC) centers in India were mentored through the Palliative Care—Promoting Assessment and Improvement of the Cancer Experience (PC-PAICE) by US and Australian academic institutions to implement a quality improvement (QI) project to improve the accessibility and quality of PC at their respective centers. The objective was to evaluate the experiences of teams in implementing QI methods across diverse geographical settings in India.

**METHODS:**

A quota sampling approach was used to elicit perspectives of local stakeholders at each site. The Consolidated Framework for Implementation Research informed development of a semistructured interview guide. Analysis leveraged deductive and inductive approaches.

**RESULTS:**

We interviewed 44 participants (eight organizational leaders, 12 clinical leaders, and 24 team members) at seven sites and identified five themes. (1) Implementing QI methods enabled QI teams to think analytically to solve a complex problem and to identify resources. (2) Developing a problem statement by identifying specific gaps in patient care fostered team collaboration toward a common goal. (3) Making use of QI tools (eg, A3 process) systematically provided a new, straightforward QI toolkit and improved QI teams' conceptual understanding. (4) Enhancing stakeholder engagement allowed shared understanding of QI team members' roles and processes and shaped interventions tailored to the local context. (5) Designing less subjective processes for patient care such as assessment scales to identify patient's symptomatic needs positively changed work practices and culture.

**CONCLUSION:**

Engaging and empowering multiple stakeholders to use QI methods facilitated the expansion and improvement of PC and cancer services in India. PC-PAICE demonstrated an efficient, effective way to apply QI methods in an international context. The impact of PC-PAICE is being magnified by developing a cadre of Indian QI leaders.

## INTRODUCTION

The epidemiology of illness in India is changing from a high prevalence of infectious ailments to a rising burden of chronic, noncommunicable diseases associated with increasing suffering at end of life.^1^ The Serious Health-related Suffering (SHS) database developed by the Lancet Commission on Global Access to Palliative Care and Pain Relief (GAPCPR) found that 7.3 million Indians were experiencing SHS in 2015. In India, people diagnosed with cancer comprised the largest and growing group of 1.3 million persons with noncommunicable diseases.^2^

**CONTEXT**

**Key Objective**
The Palliative Care—Promoting Assessment and Improvement of the Cancer Experience (PC-PAICE) project was the first quality improvement (QI) collaborative initiative between seven geographically diverse Indian health care organizations as mentee sites and US and Australian academic institutions as mentors. The purpose of this study was to evaluate the learning experiences of health care workers (such as physicians, nurses, social workers, and administrative staff) and clinical and organizational leaders after implementation of the PC-PAICE QI project.
**Knowledge Generated**
The teams learned about standard QI tools (eg, Fishbone, *Gemba walks*, and *Pareto charts*) by which to address complex problems systematically and to build a culture of QI involving stakeholders, to characterize gaps in care and identify feasible, local interventions to modify work processes and culture.
**Relevance**
The PC-PAICE international QI collaboration offers an efficient, promising model to foster shared learning and performance improvement in oncology and palliative care.

Aging is also propelling the burden of cancer in India. The Ministry of Statistics and Program Implementation projects a 41% increase in the population of Indians older than 65 years to 194 million persons, equating to 13.1% of India's population, in the coming decade.^3^ Continued aging of India's population will exacerbate the personal, family, and social burdens of chronic diseases such as cancer.^4^ Despite evidence of benefit of palliative care (PC) in serious illnesses such as cancer, in 2015, fewer than 1% of India's 1.2 billion population had access to PC.^5^

Quality improvement (QI) approaches have had promising international impacts in primary care, on prescribing practices, and for conditions other than PC in oncology.^6^ In India, QI initiatives have been implemented in the fields of cardiology, ophthalmology, and pediatrics.^7-10^ Nonetheless, bottlenecks can hinder applying QI methods in lower-resource settings and hence support and mentoring may be crucial.^11^ Several organizations have championed QI approaches in the United States to raise the bar in PC and oncology.^12^

**FIG 1:**
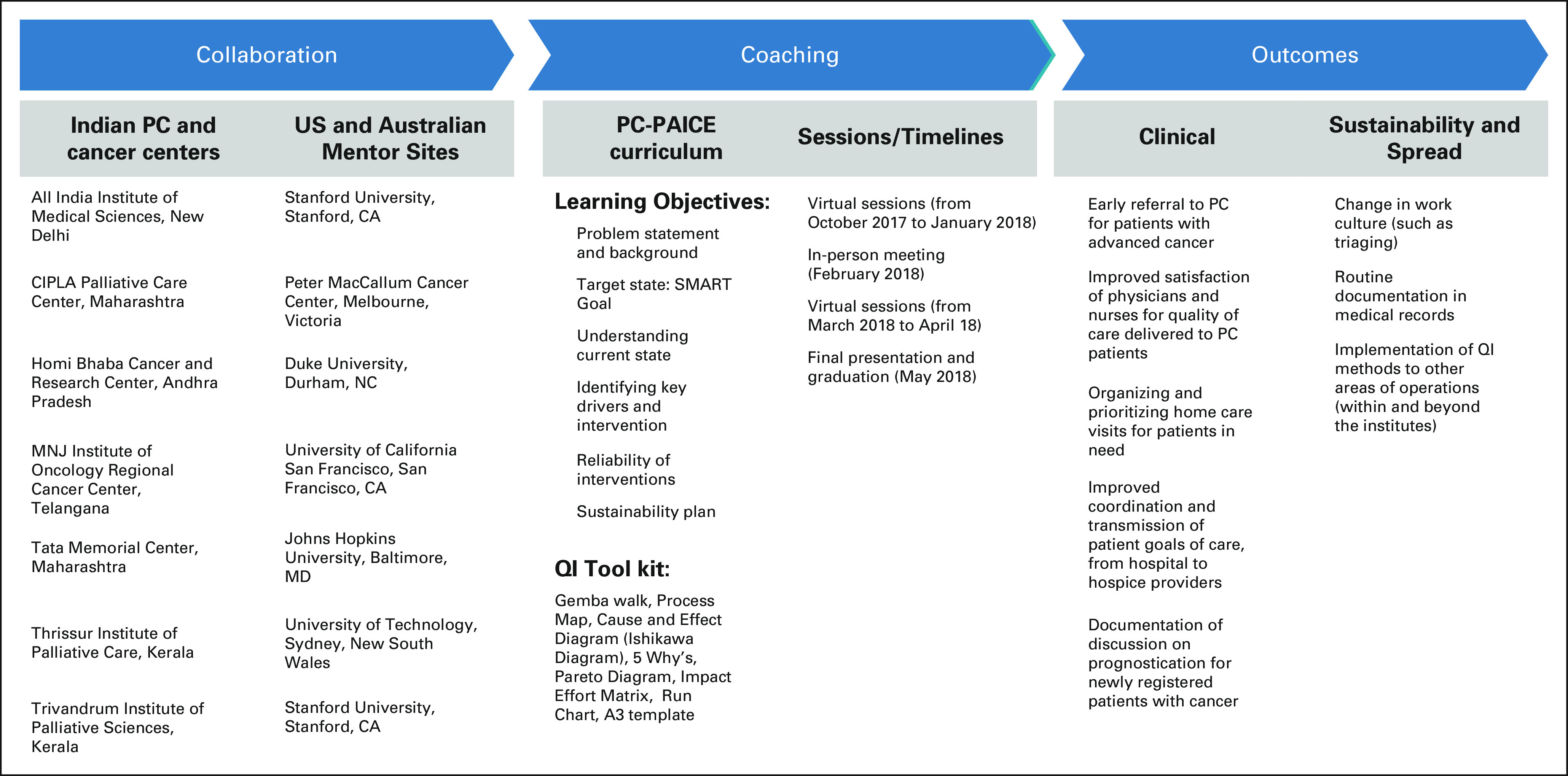
PC-PAICE framework. PC, palliative care; PC-PAICE, Palliative Care—Promoting Assessment and Improvement of the Cancer Experience; QI, Quality Improvement.

The Palliative Care: Promoting Assessment and Improvement of the Cancer Experience (PC-PAICE) remotely linked six US and Australian mentor sites with seven geographically diverse Indian mentee sites, four of which were tertiary cancer care hospitals while three were PC centers treating mainly patients with cancer. PC-PAICE aimed to improve Indian PC services through a mentored, stepwise, project-based QI learning program. Didactic content was taught in monthly Zoom sessions, and mentorship occurred in individual team meetings while projects were ongoing.^13^ The PC-PAICE program is depicted in Figure 1.

On the basis of our evaluations of cohort 1 PC-PAICE sites, we aimed to identify how the project changed the approach of QI teams to identify and tackle quality problems.

## METHODS

Each participating site had successfully implemented their QI project by the end of May 2018. The interview-based evaluation of all sites was conducted from July to September 2018. The evaluation team consisted of three PC physicians (S.B., K.A.L., and O.S.) and one qualitative methodologist (K.F.G.) who led development of the site protocol and Consolidated Framework for Implementation Research–based interview guides.^14^ At each site, we identified organizational leaders (OLs), clinical leaders (CLs), and other key QI team members (TMs) using quota sampling to ensure varying learner perspectives of the PC-PAICE experience.

After obtaining ethical approval from All India Institute of Medical Sciences (IEC-572/03/11.2017, RP-41/2017) and Stanford University (IRB-42633), 44 participants (OL-8, CL-12, TM-24) from seven Indian mentee sites were interviewed by two analysts (A.S. and A.G.) who visited five sites (sites A-E) and conducted 29 face-to-face interviews. Because of participant nonavailability and feasibility, 15 interviews were conducted via Zoom. Written informed consent was obtained from all participants during the site visit for face-to-face interviews and via email for online interviews. One of the interviewers (A.S.) was a researcher cum PC specialist while the other (A.G.) was experienced in conducting qualitative studies. Both received online training from the members of PC-PAICE analytic team (K.F.G., K.A.L., O.S., and S.B.) to conduct the interviews. One expert (K.F.G.) visited India to mentor A.S. and A.G. during two site visits. All interviews were conducted in English language except for two participants who preferred regional languages. The interviews were recorded via encrypted audio-recorder except for one participant who had requested written notes only. They were translated, transcribed, and deidentified by Indian researchers (A.S. and A.G.) and then imported into Atlas.ti version-8 for conducting analysis.^15^

A core team (K.F.G., A.G., A.S., S.C., T.H., R.C.G., and S.F.) trained in qualitative methods conducted the analyses. Two analysts (A.G. and A.S.) prepared both individual and site summaries after each interview. Three investigators (K.F.G., A.G., and A.S.) developed a preliminary codebook using closed codes (a priori) adapted from existing literature (eg, QI tools, learning, and implementation process). A final working code list was developed through constant comparison and multiple online team meetings.

The codebook was subsequently refined with open codes (a posteriori) added as new insights emerged from the data, with discrepancies rectified through continuous discussions until consensus was reached. The final code list was applied to the remaining transcripts by a single coder (A.G., A.S., S.C., T.H., R.C.G., or S.F.) and subsequently reviewed by second coder. The detailed methodology for conducting, coding, and analyzing interviews has been published.^16,17^

To inform this analysis and identify the benefits that frontline PC teams experienced from learning QI methods, we focused on the output of 339 quotes identified in transcripts that were derived from three codes *QI Methods*, *Benefits of QI Methods*, and *Benefits of the PC-PAICE Project*. The A3 problem solving template was used to guide the QI process. Standard QI tools were incorporated into the methodology to conduct root cause analyses, such as the *Gemba walk*, process mapping, cause and effect diagram (*Ishikawa diagram*), the five why's, Pareto diagram, impact effort matrix, and data monitoring using a *Run chart*.^18^ The data set was coded and reviewed by two analysts (A.S. and A.G.) to characterize the benefits of QI methods experienced by PC teams.

## RESULTS

The results illustrate how elements of the PC-PAICE QI program changed the thinking and understanding of participating teams. We present five themes that emerged as a result of learning and implementing QI methods as a part of PC-PAICE project.

### Theme 1: Implementing QI Methods Enabled QI Teams to Think Analytically to Solve a Complex Problem and to Identify Resources

Use of tools such as the *Gemba walk* and *Ishikawa diagram* enabled PC teams to better understand current care processes and causes of poor quality. A *Pareto chart* analysis helped to clarify potential causes *“which were the most significant and which were the ones that were happening most frequently”* (CL). It enabled QI teams to *“solve the problem….in a systematic way”* (TM) by breaking down the complexity of the problem and *“focus on those one or two things rather than the* *ten things”* (CL) and define achievable goals. There was an attitudinal shift for problem solving among the participants.

Similarly, tools abetted teams in systematically addressing potential solutions. Most of the interviewees agreed that QI is *“all about improving the process of workflow”* (TM) at *“workplaces using the same resources but in a different fashion and in a more systematic way”* (TM). Participants were instructed in using an Impact Effort or 2 × 2 matrix to characterize solutions and impact on *high* and *low* dimensions. This was noted by many participants as a helpful way of *“analyzing the situation”* (CL) and enabled them to focus on using available resources to most efficiently achieve meaningful impact.

As one participant summarized it, *“this is a donor driven organization (…) each time we decide to spend money a lot of discussion happens whether that section is worthwhile spending (…) whereas here (this QI project), everything was analyzed already when we asked for the funds and of course we did not look into those interventions where…, lot of input or lot of investment is required and the impact was only medium to low. We only looked at low effort, low investment and high-impact”* (CL).

As one CL summed up the QI journey, *“QI in itself kind of changes your thinking at the basic level like you don't just think of the problem, you think of the solution then you don't just arrive at the solution, you work towards the solution”* (CL).

### Theme 2: Developing a Problem Statement by Identifying Specific Gaps in Patient Care Fostered Team Collaboration Toward a Common, Clinically Meaningful Goal

Interviewees reported that most of the patients in their care presented in advanced stages of illness with no previous attention paid to their PC needs. Although TMs had experienced disappointment with poor care delivery before PC-PAICE, they had not known how to focus their efforts on improvement. As one participant noted, *“once we started doing this project, we could organize...we could actually find out who are all the people who need home care…and then after that luckily at that time we could organise home care team”* (TM).

Undertaking the QI project prompted teams to reflect deeply on both their delivery of care and the context of care, to identify gaps in patient care and in their current process. Before the QI project started, the process *“was very random and not very systematic, and very erratic...”* (TM) *“so in that what happened was, those who are needy probably would not have been attended to”* (TM). A CL stated, *“we realised that because of this ineffective handover, patient care was getting compromised (…) there is not enough information for the hospice team to continue the care...and that was a big lapse...that was existing and patient care was getting compromised...so we thought that we will take up this as a project to look at the scope and understand why things were not happening and looking at ways to improve the care transfer...”* (CL).

QI projects allowed teams to identify lacunae which were affecting clinician effectiveness. As one participant noted, *“the whole team was extremely stressed out (…) at the end of so much of hard work, despite, you know, being awake in the night, and working at least, you know, the whole day, they did not feel that they were doing best possible care for the patient. Ultimately, so there was a big discrepancy between the amount of effort put and the amount of satisfaction. So, that was an important area and that was why this problem probably was chosen by the team. They were very, very interested in improving that particular satisfaction”* (CL).

### Theme 3: Making Use of QI Tools Systematically Provided a New, Simple QI Toolkit and Improved QI Teams' Conceptual Understanding

An important contribution of PC-PAICE was to impart knowledge of the *A3 process* as a stepwise problem identification and problem-solving strategy for addressing clinical gaps. The interviewees reported that before PC-PAICE *“A3 was just a piece of paper”* (TM) and they *“could never even imagine that this could be a strategy for improving quality”* (TM). The A3 QI template provided *“whole new perspective to doing QI”* (CL) to them. They were aware of audits as a QI method, but after executing this QI project, they realized that “*QI programs can be done in this [systematic] way”* (CL).

One of the CLs whose previous experience with quality was limited to audits reported that *“audit is also a QI... but this is something different from audit... so I think this is the results that we intend to get is seen after sometime after QI projects... but in audit sometime we may think that ok we will reaudit after sometime... but this [PC-PAICE QI project] we will see the result after sometime...”* (CL).

Learning about the A3 QI process encompassed didactics and practical skills in using specific tools including the *Gemba walk*, *Ishikawa diagrams*, *Pareto chart*, *Run chart*, and other methods. These are simple tools commonly used in QI to understand cause and effect.^18^ Participants, who had limited knowledge of QI tools before the implementation of the project, could now understand and implement them in their project. A CL described *Gemba walk* *“[as] means you go to where the...where the action is happening, where the care delivery is happening and you observe what is what are the gaps in the care delivery? (…) we do the actual…we walk”* (CL). Participants further explained that *“based on that (Gemba walk) we were able to chart an Ishikawa diagram or a Fishbone diagram where we find certain...root causes or possible points where things can go wrong” *(CL).

Participants reported that they learnt new tools such as *Run chart “[as] a way to map whatever work that we are doing”* (CL). Applying these tools likewise improved their conceptual understanding of *SMART goals* and the importance of formulating a problem statement. They found QI tools to be simple, systematic, and easily applied in their settings.

### Theme 4: Enhancing Stakeholder Engagement Allowed Shared Understanding of QI Team Members' Roles and Processes, and Shaped Interventions Tailored to the Local Context

The QI project was implemented across different departments within the organization or even different organizations. Thus, it involved a variety of stakeholders such as organizational leaders, physicians, nurses, and social workers. An organizational leader emphasized that *“getting the buy-in from the individual stakeholders right at the beginning was very important”* (OL). Engaging all stakeholders during the QI project allowed shared understanding of their roles and processes and identified intervention options suitable within the local context.

Another participant noted, *“we went about by doing a group discussion and it was kind of like the initial discussion where in which we are involving the all the stakeholders who are going to make the referral to the hospice (…) involving hospice, involving hospital and then knowing that we were talking about like making them the stakeholders, making them get involved, taking their feedback, asking them what do you think of this referral form, we were going back to them. I don't think otherwise we would, I don't think that happened in the previous referral form (…) that is something this project has got in it….”* (CL).

### Theme 5: Designing Less Subjective Processes for Patient Care Such as Using Assessment Scales to Identify Patient's Symptomatic Needs Positively Changed Work Practices and Culture

One participant noted their team QI project improved early patient access to PC and even *“helped in getting (PC) awareness for the treating team and the junior doctors”* (CL). It resulted in designing new processes for patient care, such as developing and implementing assessment scales to identify patient's symptomatic needs for planning homecare visits. Redesigning existing institutional forms or patient charts improved documentation of prognosis and goals of care at end of life, *“so before doing this quality initiative project, the triaging was done on a subjective basis... but when we did this quality initiative project, we are using the tool to triage the patient... so we can easily put them into three baskets as low intensity, moderate intensity and high intensity... so that is how the triaging is happens”* (CL). As another participant noted about their project and its longer-term impact, *“We modified the hospice transfer form (…) so that the hospice team would know would be better informed about the goals of care and (…) hospice transfer form is, you know, permanent and it is ongoing”* (CL).

## DISCUSSION

We interviewed 44 participants from three different informant categories across seven geographically diverse sites in India focusing on PC for patients with cancer. We identified five major themes that elucidate how PC-PAICE participants perceived the benefits of a QI training program after they had studied and implemented QI methods to solve a quality problem in their own clinical context.

Learning QI tools and process led to an attitudinal shift and gave participants the confidence to take on a complex problem in a systematic, stepwise manner. Engaging leaders right from the beginning of the QI project helped in navigating and engaging with complex organizational hierarchies and facilitated the allocation of funds and resources necessary for implementing QI projects. Identification and involvement of all stakeholders in implementation increased teams' understanding of the complex processes and active barriers affecting multiple levels of these processes and motivated teams to take ownership of their roles and responsibilities. These findings corroborate with the observations of implementing QI projects in tertiary care pediatric settings specifically related to learning the methods of QI, stakeholder engagement, and effective resource management.^9^

QI projects have been implemented in various fields of health care in India.^7-10^ Chandra et al^8^ demonstrated that by implementing Plan-Do-Study-Act cycles, time spent in the waiting room can be reduced in ophthalmological settings. Sivanandan et al^9^ demonstrated that use of QI tools such as *Pareto charts*, Fishbone analysis, and *Run charts* improved patient outcomes in the neonatal intensive care unit. To our knowledge, this is the first time QI projects have been implemented in the oncology and PC context and have been rigorously evaluated. The PC-PAICE evaluation has demonstrated feasibility and effectiveness of QI methods to improve access to PC in resource-constrained settings. The main objective of this project was to foster capacity building for QI in India. The evidence of success and sustained collaborative activity that followed the first PC-PAICE cohort contributed to establishing QI as an ongoing feature of PC and oncology in India. After the successful outcomes of the first cohort of the PC-PAICE collaborative, two more cohorts were trained in QI methods, and a long-term QI Center, Enable Quality, Improve Patient care, India (EQuIP-India), was established by PC-PAICE leaders through a grant from the Tata Trust. Several Indian CLs from the first cohort volunteered to mentor QI projects for subsequent cohorts. Annual cohorts have continued during each year since 2017 and since 2019-2020 have included a total of 22 teams with a balanced focus on oncology and PC.^13,19-21^

This is a promising area for research which may be explored in the future QI projects. Implementing common process and outcome measures is critical to evaluating change and improvement in PC across diverse settings^22,23^ and could be a focus of strengthening India's maturing QI network. The main limitation of the present study is that constrained project resources prevented us from rigorously evaluating PC-PAICE project patient-caregiver impacts. Because of the extreme geographic spread, there was some variability in mode of interviews, with most conducted face-to-face although a few were necessarily conducted via Zoom. On the other hand, data collection was strengthened because at least one of the two interviewers and for each site was an Indian researcher familiar with local language and culture.

In conclusion, the present study demonstrates a collaborative model for learning and applying QI methods to improve PC for cancer in resource-constrained settings. Given the high burden of SHS, the growth of chronic diseases, and the lack of PC access, PC-PAICE demonstrates a model with potential to foster urgently needed, effective and sustainable improvements in patient care especially in resource-constrained settings.
